# Fusion of CCL21 Non-Migratory Active Breast Epithelial and Breast Cancer Cells Give Rise to CCL21 Migratory Active Tumor Hybrid Cell Lines

**DOI:** 10.1371/journal.pone.0063711

**Published:** 2013-05-07

**Authors:** Benjamin Berndt, Sonja Haverkampf, Georg Reith, Silvia Keil, Bernd Niggemann, Kurt S. Zänker, Thomas Dittmar

**Affiliations:** Institute of Immunology, Center for Biomedical Education and Research (ZBAF), Witten/Herdecke University, Witten, Germany; University of Illinois at Chicago, United States of America

## Abstract

The biological phenomenon of cell fusion has been linked to tumor progression because several data provided evidence that fusion of tumor cells and normal cells gave rise to hybrid cell lines exhibiting novel properties, such as increased metastatogenic capacity and an enhanced drug resistance. Here we investigated M13HS hybrid cell lines, derived from spontaneous fusion events between M13SV1-EGFP-Neo breast epithelial cells exhibiting stem cell characteristics and HS578T-Hyg breast cancer cells, concerning CCL21/CCR7 signaling. Western Blot analysis showed that all cell lines varied in their CCR7 expression levels as well as differed in the induction and kinetics of CCR7 specific signal transduction cascades. Flow cytometry-based calcium measurements revealed that a CCL21 induced calcium influx was solely detected in M13HS hybrid cell lines. Cell migration demonstrated that only M13HS hybrid cell lines, but not parental derivatives, responded to CCL21 stimulation with an increased migratory activity. Knockdown of CCR7 expression by siRNA completely abrogated the CCL21 induced migration of hybrid cell lines indicating the necessity of CCL21/CCR7 signaling. Because the CCL21/CCR7 axis has been linked to metastatic spreading of breast cancer to lymph nodes we conclude from our data that cell fusion could be a mechanism explaining the origin of metastatic cancer (hybrid) cells.

## Introduction

The biological phenomenon of cell fusion plays a fundamental role in several physiological (e.g., fertilization, placenta development, wound healing and tissue regeneration) and pathophysiological (entry of enveloped viruses and cancer) processes (for overview see: [1]). About 100 years ago the German physician Otto Aichel first hypothesized that cell fusion might be associated with tumor progression [2]. Aichel postulated that the fusion of somatic cells with tumor cells could be an explanation for chromosomal abnormalities in tumor cells [2]. Likewise, he assumed that due to fusion, tumor cells could acquire leukocyte function, such as the ability to migrate [2]. Within the past decades several studies provided evidence that fusion of two tumor cells or tumor cells and normal cells can give rise to hybrid cell lines exhibiting novel properties, such as an increased tumorigenic and metastatic behavior as well as an increased drug resistance [3]–[19]. For instance, fusion of weakly malignant Cloudman S91 melanoma cells with murine macrophages gave rise to more aggressive hybrid cells producing metastases sooner and in more mice [17]. Transplantation of human glioblastoma cells into the cheek pouch of a hamster resulted in the origin of a highly malignant and uniformly deadly for its host tumor hybrid cell line harboring both human and hamster DNA [20]. Three (CD74, CXCR4, PLAGL2) of 7 human genes found in these tumor/hamster hybrid cells showed transcriptional activities *in vivo* or *in vitro* for years [8]. Because all of them are implicated with malignancy of glioblastoma these data support the thesis that genetic hybridization of cancer and normal cells can transmit malignancy [8]. This is in view with data demonstrating that malignant breast cancer epithelial cells spontaneously fuse and transform mouse stroma cells, thereby giving rise to hybrid cells of which some possessed a mixed human and mouse karyotype including mouse/human translocations [21]. Recent data of Wang et al. suggested that the spontaneous fusion between prostate cancer cells and prostate stroma cells could be a mechanism of prostate cancer androgen-independent progression [22]. By applying a parabiosis model in which a GFP mouse was surgically joined to an APC^Min/+^:ROSA26 mouse the authors were able to identify GFP and β-galactosidase double positive cells in the transformed intestinal tissue of the APC^Min/+^:ROSA26 mouse indicating that cell fusion has occurred [14]. Isolation of these hybrid cells and subsequent transcriptome analysis showed identity characteristics of both parental derivatives, but also showed a unique subset of transcriptomes including genes known to be modulated in metastasis [14].

In a previous study we have shown that breast epithelial cells exhibiting stem cell properties spontaneously fuse with breast cancer cells, thereby giving rise to hybrid cell lines exhibiting novel properties, such as an altered migratory activity and an enhanced drug resistance [4], [5], [23]. Flow cytometric analysis of M13HS-2 and M13HS-8 hybrid cell lines demonstrated expression of the chemokine receptor CCR7 [5], which is a member of the seven transmembrane G protein-coupled receptor family that has two ligands: CCL19 and CCL21 [24]. CCL19 is expressed by lymphatic endothelial cells, whereas CCL21 is constitutively expressed on specialized high endothelial venules (HEVs) of lymph nodes, Peyer's patches, thymus, spleen and mucosal tissue [25], [26]. CCR7 is prevalent in various subsets of T lymphocytes and activated dendritic cells and the interaction with its ligand CCL21 recruits these cell populations to the lymph nodes [24], [25].

In accordance to other G protein-coupled receptors CCR7 activates signal transduction via G protein-dependent and independent mechanisms, whereby CCL19 and CCL21 elicit different cellular functions in various cell types (for review see [27]). For instance, both ligands induce G protein activation and calcium mobilization, indicating PLC-β/γ activation, with equal potency, but only activation by CCL19, but not CCl21, promotes robust desensitization of endogenous CCR7 due to receptor phosphorylation and β-arrestin recruitment in a human T cell lymphoma cell line [28]. The differential effects of both ligands on CCR7 signaling and desensitization might be attributed to striking differences in activation of the G protein-coupled receptor (GRK)/β-arrestin system [29]. CCL19 dependent β-arrestin2 recruitment is catalyzed by both GRK3 and GRK6, whereas CCL21 activates GRK6 alone indicating that GRK3 activity is involved in CCR7 desensitization [29]. In dendritic cells CCL19/CCL21 mediated CCR7 G protein-dependent signaling leads to activation of MAPK family members ERK1/2 (MAPK^p42/44^), p38, and JNK as well as PI3K (for review see [27]). In CD4 T-cells CCL21 modulates T-cell receptor signaling through Ras and Rac dependent pathways concomitant with increased phosphorylation levels of AKT, MEK, and MAPK^p42/44^ [30]. Interestingly, neither p38 nor JNK were phosphorylated in CCL21 co-stimulated CD4 T-cells [30] indicating CCR7 specific signal transduction cascades vary among different cell types. Likewise, a linkage of G protein-coupled receptors to the MAPK signaling pathway through class IB PI3Kγ and phosphotyrosine kinase (PTK), SHC, GRB2, SOS, RAS and RAF signaling has been reported [31]. In contrast to G protein dependent signaling, MAPK activation is also facilitated via a G protein-independent mechanisms due to CCL19/CCL21 mediated engagement of GRK6/β-arrestin 2 [29], which may point to a pivotal role of MAPK activity in CCR7 signaling.

Analysis of HEK293 CCR7 expressing cells demonstrated calcium mobilization, MAPK^p42/44^ and FAK phosphorylation and induction of cell migration upon CCL21 stimulation [32]. A CCR7 mediated PI3K and PLCγ dependent invasive phenotype independent of EGFR signaling has been reported for squamous cell carcinoma of the head and neck (SCCHN) [33].

In the context of cancer CCR7 expression of tumor cells has been associated with lymph node metastasis of various tumors, including breast (for review see: [34]). Analysis of breast cancer and lymph nodes tissue microarrays of the same patients revealed that CXCR4 and CCR7 together with their ligands as well as EGFR were significantly higher expressed in tumor cells with lymph node metastasis [35]. Moreover, Kaplan-Meier survival analysis showed that patients exhibiting high CXCR4, CCR7, and EGFR expression experienced a shorter survival period compared with those with low expression suggesting that these receptors can serve as an indicator of undesirable prognosis in patients with breast cancer [35]. In addition to induction of a migratory phenotype [33] and directing metastasizing tumor cells to regional lymph nodes [34], [35] the CCR7/CCL21 axis may also foster tumor metastasis by preventing anoikis in cancer cells [36] and up-regulation of matrix metalloproteinase-9 (MMP9) [37], [38].

Because cell fusion has been linked to cancer metastasis [6], [10], [13] and M13HS-2 and M13HS-8 were positive for CCR7 expression we thus investigated whether the CCL21/CCR7 axis could be activated in these hybrid cell lines. Our data show that both hybrid cell lines responded to CCL21 stimulation with an increased migratory activity. Because the migratory behavior of parental derivatives remained unaltered in the presence of CCL21 despite CCR7 expression our data provide evidence that cell fusion can give rise to hybrid cell lines exhibiting novel properties.

## Materials and Methods

### Cell culture

Cells were cultured as described previously [5]. M13SV1-EGFP-Neo breast stem cells exhibiting stem cell characteristics were generated by stable transfection of M13SV1 cells (kindly provided by James E. Trosko, Michigan State University, Lansing; [39]) with pEGFP-MCS-Neo [40]. Cells were maintained in MSU-1 basal media (Sigma-Aldrich, Taufkirchen, Germany) supplemented with 10% FCS (PAA Laboratories, Linz, Austria), 1% Penicillin/Streptomycin (100 U/ml Penicillin, 0.1 mg/ml Streptomycin; PAA Laboratories, Linz, Austria), 10 µg/ml human recombinant EGF, 5 µg/ml human recombinant insulin, 0.5 µg/ml hydrocortisone, 4 µg/ml human transferrin, 10 nM β-estradiol (all supplements were purchase from Sigma-Aldrich, Taufkirchen, Germany), and 400 µg/ml G418 (Biochrom AG, Berlin, Germany). HS578T-Hyg breast cancer cells were generated by stable transfection of HS578T cells (HTB-126; LGC Standards GmbH, Wesel, Germany) with pKS-Hyg. Cells were cultured in RPMI 1640 media (PAA Laboratories, Linz, Austria) supplemented with 10% FCS (PAA Laboratories, Linz, Austria), 1% Penicillin/Streptomycin (PAA Laboratories, Linz, Austria) and 200 µg/ml Hygromycin B (PAA Laboratories, Linz, Austria). M13HS-2 and M13HS-8 hybrid cell lines derived form spontaneous fusion events between M13SV1-EGFP-Neo cells and HS578T-Hyg cells [5] and were maintained in RPMI 1640 media (PAA Laboratories, Linz, Austria) supplemented with 10% FCS (PAA Laboratories, Linz, Austria), 1% Penicillin/Streptomycin (PAA Laboratories, Linz, Austria), 200 µg/ml Hygromycin B (PAA Laboratories, Linz, Austria), and 400 µg/ml G418 (Biochrom AG, Berlin, Germany). All cells were kept at 37°C and 5% CO_2_ in a humidified atmosphere.

### Western Blot analysis

CCR7, AKT, and MAPK^p42/44^ expression as well as AKT and MAPK^p42/44^ phosphorylation levels were determined by Western Blot analysis. For detection of CCR7 expression cells (1×10^6^) were lysed in modified RIPA buffer (150 mM NaCl, 50 mM Tris-HCl pH 7.4, 1 mM EDTA, 1 mM PMSF, 1% Triton X-100, 1% sodium deoxycholate acid, 0.1% SDS, 5 µg/ml aprotinin, 5 µg/ml leupeptin; all chemicals were purchased from Sigma-Aldrich, Taufkirchen, Germany). Cell debris was removed by centrifugation and the cell lysate containing supernatant was boiled for 5 minutes in 3× Laemmli Sample Buffer [41]. To investigate AKT and MAPK^p42/44^ phosphorylation cells (2×10^5^ per sample) were kept in suspension and were treated with 500 ng/ml CCL21 for 0 min, 1 min, 2 min, 5 min and 10 min. In dependence of blocking PI3K/AKT as well as MAPK signaling cells (2×10^5^ per sample) were first pre-treated with 500 nM Ly294002 (IC_50_  =  1.4 µM; VWR International GmbH, Darmstadt, Germany) for 60 min or 500 nM PD98059 (MEK1: IC_50_  =  4 µM MEK2: IC_50_  =  50 µM; VWR International GmbH, Darmstadt, Germany) for 30 min, respectively, and then stimulated with 500 ng/ml CCL21 for 5 min. Samples were lysed in 3× Laemmli Sample Buffer (5 minutes, 95°C) [41].

Samples were separated by SDS-PAGE on a 10% SDS polyacrylamide gel and transferred to PVDF nitrocellulose membranes (Millipore, Schwalbach, Germany) under semi-dry conditions. Membranes were blocked overnight with 10% (w/v) non-fat dry milk powder in TBS-T. The following antibodies were used in this study: CCR7 (rabbit polyclonal, antibodies-online GmbH, Aachen, Germany), AKT (clone 11E7; rabbit monoclonal, Cell Signalling, New England Biolabs, Frankfurt am Main, Germany), pAKT S473 (clone D9E; rabbit monoclonal, Cell Signalling; New England Biolabs, Frankfurt am Main, Germany), MAPK^p42/44^ (rabbit polyclonal; Cell Signalling, New England Biolabs, Frankfurt am Main, Germany), pMAPK^p42/44^ (rabbit polyclonal; Cell Signalling, New England Biolabs, Frankfurt am Main, Germany). Elf4E (rabbit polyclonal; Cell Signalling, New England Biolabs, Frankfurt am Main, Germany) served as a loading control. For detection of primary antibodies the HRP-conjugated secondary anti-rabbit IgG (Cell Signalling, New England Biolabs, Frankfurt am Main, Germany) was used. Bands were visualized using the LumiGLO® Reagent (Cell Signalling, New England Biolabs, Frankfurt am Main, Germany) in accordance to the manufacturers' instructions and were detected with the Aequoria Macroscopic Imaging system (Hamamatsu Photonics Germany, Herrsching am Ammersee, Germany). Densitometric analysis of Western Blot results was performed by using the Image J (http://rsb.info.nih.gov/ij/) software application. Relative intensities of native and phosphorylated proteins were determined in relation to the housekeeping gene elf4E, whereby untreated cells served as a control and were set to 100%.

### Flow cytometry

Flow cytometry was performed on a FACScalibur floy cytometer (Becton Dickenson, Heidelberg, Germany). Changes in the intracellular calcium levels of cells were determined by a flow cytometry-based measurement in accordance to Gergely et al. [42] as described previously [23], [43]–[45]. In brief, cells (5×10^5^) were labeled with 4 µM Fluo-3 (Invitrogen, Karlsruhe, Germany) prior to stimulation with CCL21 (500 ng/ml; Biotrend GmbH, Cologne, Germany), U73122 (5 µM; IC_50_  =  1 – 5 µM; VWR International GmbH, Darmstadt, Germany), or a combination of both. Cells were pretreated with 5 µM U73122 prior to measurements. CCL21 was added after 50 s. Subsequently, the tube was mixed and acquisition was continued for a total of 204.80 s. For analysis, the mean fluorescence intensity (MFI) of 10 s intervals was determined, whereby the MFI of the first 50 s of each calcium measurement without stimulus was defined as the baseline level (MFI_baseline_). This value was compared to the MFI of the first 60 s after stimulation (MFI_stimulation_). The MFI_stimulation_ of each calcium measurement was calculated in relation to the appropriate MFI_baseline_ of the same experiment, which was set to 100%. Flow cytometry data were analyzed using the WinMDI 2.8 software (http://www.methods.info/software/flow/winmdi.html).

### Cell migration studies

To investigate the migratory properties of cells in response to CCL21 the 3D-collagen matrix migration assay was applied as described previously [5], [23], [43], [46]. In brief, liquid collagen solution (Purecol; Nutacon BV, Leimuiden, The Netherlands) was mixed with 10× MEM (Sigma-Aldrich, Taufkirchen, Germany), 7.5% sodium bicarbonate solution (Sigma-Aldrich, Taufkirchen, Germany), and cells (6×10^4^). In dependence of the experimental setting CCL21 (500 ng/ml; Biotrend GmbH, Cologne, Germany), 5 µM U73122 (IC_50_  =  1 – 5 µM; VWR International GmbH, Darmstadt, Germany), 500 nM Ly294002 (IC_50_  =  10 µM; VWR International GmbH, Darmstadt, Germany), or 500 nM PD98059 (MEK1: IC_50_  =  4 µM MEK2: IC_50_  =  50 µM; VWR International GmbH, Darmstadt, Germany) were added to the solution. The collagen-cell suspension was filled in self-constructed cell migration chambers and the collagen was allowed to polymerize. Subsequently, cell migration chambers was put under a microscope into wooden boxes being tempered to 37°C. Cell migration was recorded for at least 15 h by time-lapse video microscopy. For data analysis, 30 cells of each sample were randomly selected and two-dimensional projections of the paths of the migrating cells were digitized in 15 min intervals.

### CCR7 siRNA experiments

Cells (1×10^6^) were transfected with 450 nM ON-TARGET plus SMART pool Human CCR7 siRNA (Thermo Fischer Scientific Dharmacon, Epsom, United Kingdom) or control siRNA (AllStars Negative Control siRNA, Qiagen, Hilden, Germany) by electroporation using the Amaxa Nucleofector™ II (Lonza, Cologne, Germany) in accordance to the manufacturers' instructions. Downregulation of CCR7 expression was determined by Western-Blot analysis.

## Results

### M13HS-2 and M13HS-8 hybrid cell lines possess a functional CCR7 signaling

**Figure 1:**
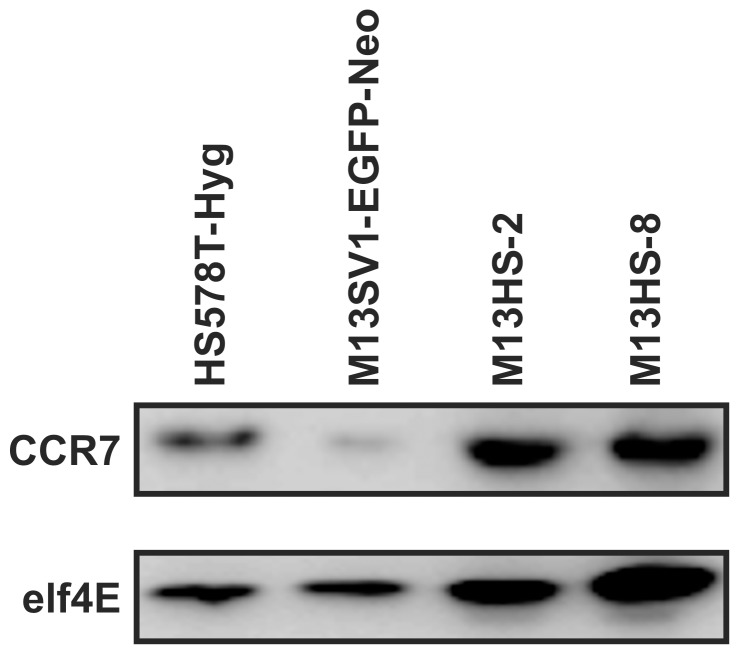
CCR7 expression of parental cell lines and hybrid cell lines. CCR7 expression was detected by Western Blot analysis, whereby elf4E expression was used as an internal control. Shown are representative Western Blot data of n = 3 independent experiments.

**Figure 2:**
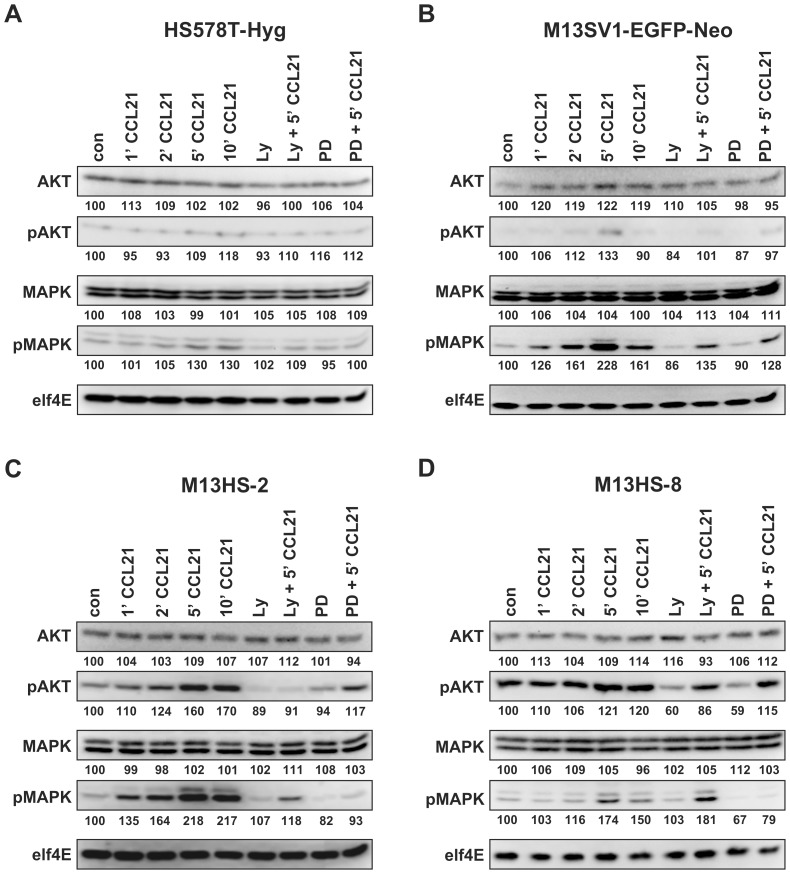
Analysis of signal transduction cascades initiated by CCL21/CCR7 signaling. Cells were treated with 500 ng/ml CCL21 for 0, 1, 2, 5, and 10 minutes. For inhibitor studies cells were preincubated for 60 minutes with 500 nM Ly29004 or 30 minutes with 500 nM PD98059, respectively. Cells were analyzed for AKT phosphorylation and MAPK^p42/44^ (MAPK) phosphorylation, whereby elf4E was used as a housekeeping gene. Values indicate the relative intensities of proteins in relation to elf4E, which was set to 100%. Shown are representative Western Blot data of n = 3 independent experiments. **(A)** HS578T-Hyg breast cancer cells, **(B)** M13SV1-EGFP-Neo breast epithelial cells exhibiting stem cell characteristics, **(C)** M13HS-2 hybrid cells, **(D)** M13HS-8 hybrid cells.

M13HS-2 and M13HS-8 hybrid cell lines, which derived from spontaneous fusion events between M13SV1-EGFP-Neo human breast epithelial cells exhibiting stem cell characteristics and HS578T-Hyg human breast cancer cells, express the CCL21 receptor CCR7 (Figure 1). The CCR7 expression level of HS578T-Hyg breast cancer cells was similar to hybrid cell lines, whereas M13SV1-EGFP-Neo cells exhibited a weaker CCR7 expression (Figure 1). Because the interaction of CCL21 with its receptor CCR7 has been linked to lymph node metastasis of breast cancer and other tumor types (for review see: [34]), we first investigated whether hybrid cell lines respond to CCL21 with an activation of CCR7 specific signal transduction cascades including PI3K/AKT, MAPK^p42/44^ and PLC-β/γ signaling [27]. Suspension cells were stimulated for 1, 2, 5, and 10 minutes with 500 ng/ml CCL21 as well as Ly294002 (PI3K inhibitor; 500 nM) and PD98059 (MEK inhibitor; 500 nM) and subsequently analyzed for AKT and MAPK^p42/44^ phosphorylation (Figure 2). In addition to Figure 2 a densitometric analysis of Western Blot data has been performed, which is provided as supporting information (Figure S1).

Stimulation of HS578T-Hyg breast cancer cells with CCL21 did not result in AKT phosphorylation (Figure 2A). By contrast, a slight MAPK^p42/44^ phosphorylation was observed with a peak maximum after 5 minutes of CCL21 stimulation (Figure 2A). As expected, treatment of HS578T-Hyg cells with the PI3K inhibitor Ly294002 did not alter pAKT levels, whereas treatment with PD98059 resulted in decreased pMAPK^p42/44^ levels in CCL21 stimulated cells (Figure 2A). Interestingly, we also noticed decreased pMAPK^p42/44^ levels in Ly294002 plus CCL21 stimulated HS578T-Hyg cells (Figure 2A), which may point to a possible cross-talk between PI3K/AKT signaling and MAPK^p42/44^ signaling.

Stimulation of M13SV1-EGFP-Neo breast epithelial cells exhibiting stem cell characteristics with CCL21 indicated activation of PI3K/AKT and MAPK^p42/44^ signaling with a peak maximum after 5 minutes (Figure 2B). However, compared to MAPK^p42/44^ phosphorylation activation of PI3K/AKT signaling was rather moderate in this cell line as indicated by faint pAKT levels (Figure 2B). Treatment of M13SV1-EGFP-Neo cells with Ly294002 and PD98059 resulted in decreased AKT and MAPK^p42/44^ phosphorylation levels (Figure 2B). Interestingly, inhibition of PI3K activity by Ly294002 also impaired MAPK^p42/44^ phosphorylation (Figure 2B), which is similar to HS578T-Hyg breast cancer cells (Figure 2A). Conjointly, treatment of M13SV1-EGFP-Neo cells with PD98059 also caused decreased pAKT levels (Figure 2B). Again, these data suggest that a putative crosstalk between PI3K/AKT signaling and MAPK signaling do also exist in M13SV1-EGFP-Neo breast epithelial cells exhibiting stem cell properties.

M13HS-2 and M13HS-8 hybrid cell lines revealed a similar CCL21 mediated AKT and MAPK^p42/44^ phosphorylation pattern (Figure 2C,D). Cells showed a time-dependent increased AKT and MAPK^p42/44^ phosphorylation with a peak maximum after 5 to 10 minutes (Figure 2C,D). However, compared to M13HS-2 hybrid cells the CCL21 mediated AKT phosphorylation was rather moderate in the M13HS-8 hybrid cell line, which might be attributed to the fact that M13HS-8 cells possessed higher basal pAKT levels (Figure 2D). Co-treatment of M13HS-2 hybrid cells with Ly294002 and CCL21 as well as PD98059 and CCL21 resulted in decreased pAKT and pMAPK^p42/44^ levels (Figure 2C). Moreover, Ly294002 plus CCL21 also impaired MAPK^p42/44^ phosphorylation, whereas pAKT levels were also decreased in PD98059 plus CCL21 treated M13HS-2 hybrid cells (Figure 2C), which is similar to M13SV1-EGFP-Neo breast epithelial cells exhibiting stem cell properties (Figure 2B) and which suggests a putative active crosstalk between PI3K/AKT signaling and MAPK^p42/44^ signaling.

Co-treatment of M13HS-8 hybrid cells with Ly294002 and CCL21 as well as PD98059 and CCL21 also resulted in decreased pAKT and pMAPK^p42/44^ levels (Figure 2D). However, in contrast to M13HS-2 hybrid cells, the PI3K inhibitor Ly294002 did not impair MAPK^p42/44^ phosphorylation in M13HS-8 cells (Figure 2D). By contrast, treatment of M13HS-8 hybrid cells only decreased basal pAKT levels, but did not alter CCL21 induced AKT phosphorylation (Figure 2D).

In summary, all tested cell lines responded to CCL21 stimulation with activation of CCR7 specific signal transduction cascades, but differ in the intensity and kinetics of these signal cascades.

### Calcium measurements

**Figure 3:**
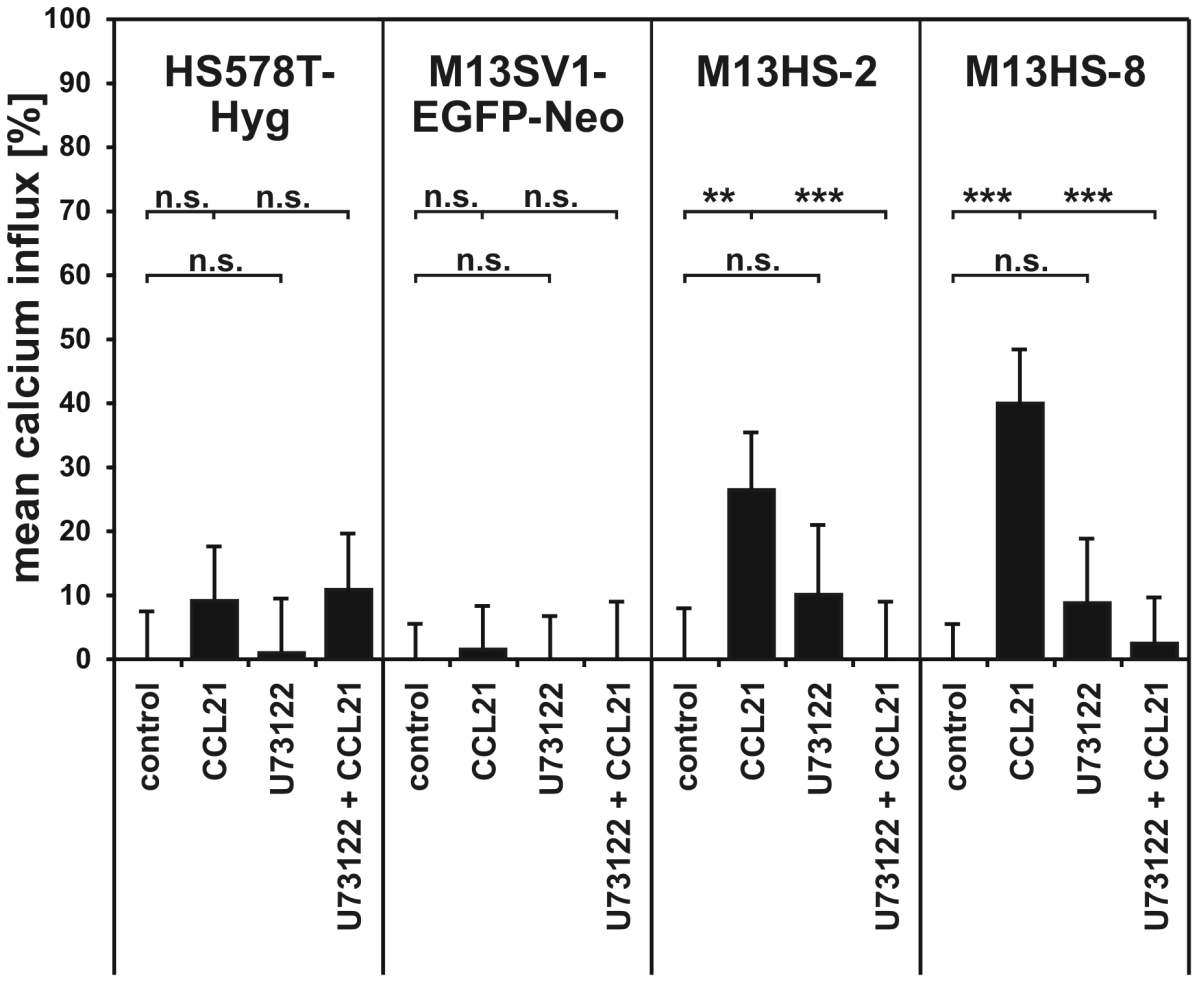
Calcium measurements. The diagram shows the mean calcium influx in CCL21 stimulated cells. An increase in cytosolic calcium levels was only observed in M13HS-2 and M13HS-8 hybrid cells. To prove whether the CCL21 induced calcium influx in these cell lines depended on PLC-β/γ activity, appropriate calcium measurements within the presence of 5 µM U73122 were performed. Cells were preincubated for 5 minutes with U73122 prior to analysis. Shown are the mean±SD of n = 3 independent experiments. Statistical significance was calculated using Student's *t*-test: n.s.  =  not significant; **  =  *p*<0.01; ***  =  *p*<0.001.

In addition to Western Blot studies analyzing PI3K/AKT and MAPK signaling we also performed flow cytometry based calcium measurements [23], [43]–[45] to investigate whether the CCL21/CCR7 axis does also active PLC-β/γ signaling. Flow cytometry based calcium measurements revealed no significant CCL21 mediated calcium influx in M13SV1-EGFP-Neo and HS578T-Hyg parental cells (Figure 3). By contrast, both hybrid cell lines showed significantly increased intracellular calcium levels upon CCL21 stimulation indicating activation of PLC-β/γ signaling (M13HS-2 control: 0±8% vs. 500 ng/ml CCL21: 26±9%, p<0.01; M13HS-8 control: 0±5% vs. 500 ng/ml CCL21: 40±6%, p<0.001; Figure 3). This was further supported by addition of the PLC-β/γ inhibitor U73122 that completely abolished the CCL21 mediated calcium influx in M13HS-2 and -8 hybrid cell lines (M13HS-2: 500 ng/ml CCL21: 26±9% vs. 5 µM U73122 + 500 ng/ml CCL21: 0±9%, p<0.001; M13HS-8: 500 ng/ml CCL21: 40±6% vs. 5 µM U73122 + 500 ng/ml CCL21: 3±4%, p<0.001; Figure 3).

### M13HS-2 and M13HS-8 hybrid cell lines respond to CCL21 with an increased migratory activity

**Figure 4:**
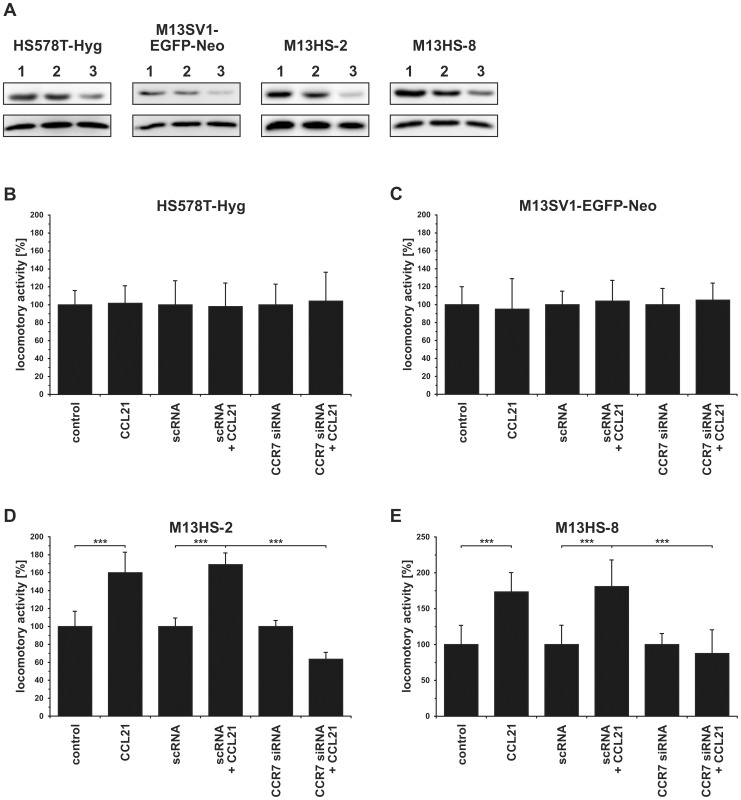
Knockdown of CCR7 expression abolish the CCL21 induced migration of M13HS hybrid cell lines. The migratory activity was analyzed using the 3D collagen matrix migration assay combined with time-lapse video-microscopy. For a better comparison the locomotory activities of CCL21 stimulated cells were calculated in relation to untreated control cells, which were set to 100%. Cells were stimulated with 500 ng/ml CCL21. Shown are the mean±SD of n = 3 independent experiments. **(A)** HS578T-Hyg breast cancer cells, **(B)** M13SV1-EGFP-Neo breast epithelial cells exhibiting stem cell characteristics, **(C)** M13HS-2 hybrid cells, **(D)** M13HS-8 hybrid cells. Statistical significance was calculated using Student's *t*-test: ***  =  *p*<0.001.

Because the CCL21/CCR7 axis has been associated with lymph node metastasis of breast cancer (for review see: [34]) cell migration studies were conducted by using the 3D-collagen matrix migration assay. In addition to naïve cells cell migration studies were also performed with cells either transfected with specific CCR7 siRNA or scrambled control RNA (scRNA). CCR7 expression data are summarized in Figure 4A and show a reduced CCR7 expression in CCR7 siRNA transfected cells, whereas CCR7 levels of scRNA transfected cells were comparable to naïve cells (Figure 4A).

Both parental cell lines did not show an altered migratory activity in the presence of CCL21 (Figure 4B,C). By contrast, the locomotory activity of both M13HS-2 and M13HS-8 hybrid cell lines was markedly increased upon CCL21 stimulation (M13HS-2 500 ng/ml CCL21: 160±23%, p<0.001; M13HS-8 500 ng/ml CCL21: 173±27%, p<0.001; Figure 4D,E). Knockdown of CCR7 expression by siRNA had no impact on the migratory activity of parental M13SV1-EGFP-Neo breast epithelial cells exhibiting stem cell properties and HS578T-Hyg breast cancer cells as compared to naïve and scRNA transfected cells (Figure 4B,C). By contrast, knockdown of CCR7 expression in M13HS-2 and M13HS-8 hybrid cell lines resulted both in a significantly decreased locomotory activity (M13HS-2 scRNA + CCL21: 169±13% vs. CCR7 siRNA + CCL21: 63±8%; p<0.001; M13HS-8 scRNA + CCL21: 181±37% vs. CCR7 siRNA + CCL21: 83±29%; p<0.01; Figure 4D,E). These data indicate that the CCL21 mediated induction of M13HS-2 and M13HS-8 cell migration is mediated via CCR7 signaling.

### Differential engagement of CCR7 signal transduction cascades in the CCL21 induced migration of hybrid cell lines

Western Blot data and calcium measurements revealed a differential engagement of signal transduction cascades in M13HS hybrid cell lines in comparison to their parental derivatives. To investigate the influence of PLC-β/γ, PI3K/AKT, and MAPK signaling in the CCL21 induced migration of M13HS-2 and -8 hybrid cell lines the migratory activity of the cells was measured in the presence of specific inhibitors (U73122: 5 µM; Ly294002: 500 nM; PD98059: 500 nM) targeting the above mentioned signal transduction cascades.

**Figure 5:**
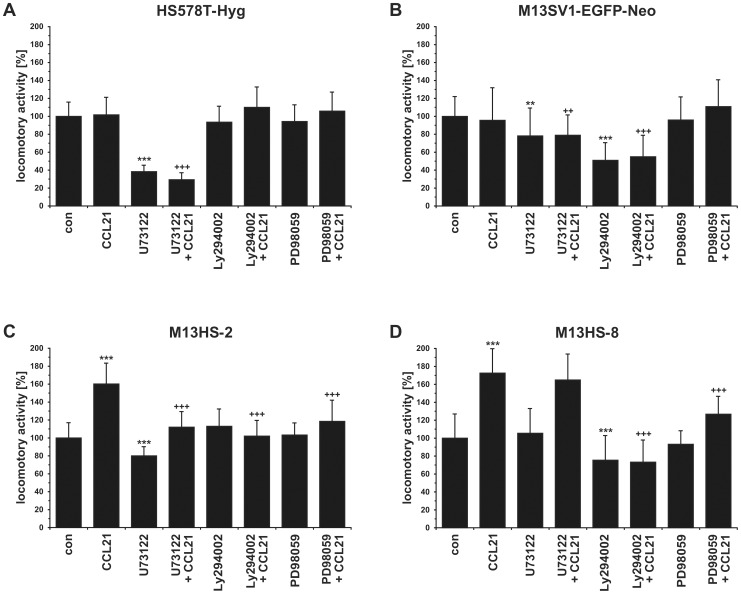
The CCL21 induced migration of M13HS hybrid cell lines depends on a different signal transduction cascades. The migratory activity was analyzed using the 3D collagen matrix migration assay combined with time-lapse video-microscopy. For a better comparison the locomotory activities of CCL21 and inhibitor stimulated cells were calculated in relation to untreated control cells, which were set to 100%. Cells were treated with 500 ng/ml CCL21, 5 µM U73122, 500 nM Ly294002, and 500 nM PD98059. Shown are the mean±SD of n = 4 independent experiments. **(A)** HS578T-Hyg breast cancer cells **(B)** M13SV1-EGFP-Neo breast epithelial cells exhibiting stem cell characteristics, **(C)** M13HS-2 hybrid cells, **(D)** M13HS-8 hybrid cells. Statistical significance was calculated using Student's *t*-test: “*”  =  statistical significance vs. control: **  =  *p*<0.01; ***  =  *p*<0.001. “+”  =  statistical significance vs. CCL21: ++  =  *p*<0.01; +++  =  *p*<0.001.

Analysis of HS578T-Hyg cells showed a marked inhibitory effect of U73122 on the cells migratory activity, which was similar to U73122 plus CCL21 treated cells (U73122: 38±7%; p<0.001; U73122 + CCL21: 29±8%; p<0.001; Figure 5A) indicating that the matrix induced migration of HS578T-Hyg breast cancer cells depends on PLC-β/γ signaling. By contrast, treatment of HS578T-Hyg breast cancer cells with the PI3K inhibitor Ly294002 did not alter the cells migratory activity (both control and CCL21 treated cells) (Figure 5A), which is in view with Western Blot studies showing no AKT phosphorylation in CCL21 stimulated HS578T-Hyg cells (Figure 2A). Likewise, inhibition of MAPK^p42/44^ signaling with PD98059 had no effect on the migration of both control and CCL21 stimulated HS578T-Hyg breast cancer cells albeit cells displayed increased MAPK^p42/44^ levels upon CCL21 stimulation (Figure 2A). Moreover, because the PI3K inhibitor Ly294002 also blocked the CCL21 induced MAPK^p42/44^ phosphorylation in HS578T-Hyg cells (Figure 2A), but did not alter the cells migratory behavior these data suggest that MAPK^p42/44^ signaling is not involved in the migration of HS578T-Hyg breast cancer cells.

Analysis of M13SV1-EGFP-Neo breast epithelial cells exhibiting stem cell characteristics showed that the migratory activity of the untreated and CCL21 stimulated cells was decreased in the presence of both U73122 (U73122: 78±31%; p<0.01; U73122 + CCL21: 78±23%; p<0.01; Figure 5B) and Ly294002 (Ly294002: 55±20; p<0.001; Ly294002 + CCL21: 55±24%; p<0.001; Figure 5B. By contrast, blockade of MAPK^p42/44^ signaling with PD98059 did not alter the cells locomotory behavior (Figure 5B). These data indicate that the matrix-induced migration of the M13SV1-EGFP-Neo cells depends on PLC-β/γ and PI3K/AKT, but not on MAPK signaling.

Western Blot studies revealed that Ly294002 also inhibited MAPK^p42/44^ phosphorylation in M13SV1-EGFP-Neo and that PD98059 blocked AKT phosphorylation in CCL21 stimulated M13SV1-EGFP-Neo cells, respectively (Figure 2B), suggesting a crosstalk between PI3K and MEK/MAPK signaling and vice versa. However, M13SV1-EGFP-Neo cell migration predominantly depends on PI3K/AKT signaling, but not MAPK^p42/44^ signaling. Thus, even though these crosstalks seems to be active in M13SV1-EGFP-Neo cells they are not involved in the cells migratory behavior. Otherwise, a PD98059 dependent effect should have been observed.

M13HS-2 and M13HS-8 hybrid cell lines responded differently to the PLC-β/γ inhibitor U73122. Both the matrix-induced and CCL21 induced migration of M13HS-2 hybrid cells was blocked by U73122 (U73122: 80±9%; p<0.001; U73122 + CCL21: 112±16%; p<0.001; Figure 5C) indicating the necessity of PLC-β/γ activity in directing M13HS-2 cell migration. By contrast, inhibition of PLC activity by U73122 neither altered the spontaneous nor the CCL21 mediated migratory activity of M13HS-8 cells (Figure 5D) albeit calcium measurements demonstrated a CCL21 induced calcium influx in M13HS-8 hybrid cell lines (Figure 3). Thus, PLC-β/γ signaling is not involved in the spontaneous and the CCL21 induced migration of M13HS-8 cells.

Treatment of M13HS-2 hybrid cells with the PI3K inhibitor Ly294002 and the MEK inhibitor PD98059 significantly blocked the CCL21 induced cell migration of the cells, but had no effect on the spontaneous migration of the cells (Ly294002: 112±19%; n.s.; Ly294002 + CCL21; p<0.001; PD98059: 103±13%; n.s.; PD98059 + CCL21: 119±24%: p<0.001; Figure 5C). Migration data of M13HS-2 hybrid cells are in view with Western Blot data demonstrating a CCL21 dependent activation of PI3K/AKT and MAPK signaling (Figure 2C). Western Blot data further revealed that Ly294002 impaired MAPK^p42/44^ phosphorylation and that PD98059 inhibits AKT phosphorylation in CCL21 stimulated M13HS-2 hybrid cells. Because the inhibitory effect was nearly similar for both inhibitors it may be speculated that each inhibitor impaired the CCL21 induced migration of M13HS-2 by blocking both PI3K/AKT and MAPK signaling. By contrast, in untreated M13HS-2 hybrid cells each inhibitor specifically blocked its target molecule (and pathway) (Figure 2C). For instance, pAKT levels were decreased in Ly294002 treated M13HS-2 cells, but pMAPK^p42/44^ levels remained unaltered as compared to control cells (Figure 2C). Thus, even if one signal transduction pathway (e.g., PI3K/AKT) is blocked in M13HS-2 hybrid cells, intracellular processing of cell migration signals induced by the surrounding matrix could be facilitated by another signal transduction pathway (e.g., MAPK).

In contrast to M13HS-2 hybrid cells Ly294002 significantly blocked both the spontaneous and the CCL21 induced migration of M13HS-8 hybrid cells (Ly294002: 75±27%; p<0.001; Ly294002 + CCL21: 73±25%; p<0.001; Figure 5D), which might be attributed to the fact that M13HS-8 hybrids already exhibited markedly elevated basal pAKT levels that are effectively blocked by Ly294002 (Figure 2D). In accordance to M13HS-2 hybrid cells inhibition of MAPK^p42/44^ activity by PD98059 only blocked the CCL21 induced, but not the spontaneous migration of M13HS-8 hybrid cells (PD98059: 93±15%; n.s.; PD98059 + CCL21: 127±20%; p<0.001; Figure 5D). PD98059 effectively impaired MAPK^p42/44^ phosphorylation in untreated and CCL21 stimulated M13HS-8 cells (Figure 2D), which is similar to M13HS-2 cells. By contrast, treatment with PD98059 resulted in decreased pAKT levels in untreated M13HS-8 hybrid cells, whereas pAKT levels of PD98059 plus CCL21 co-treated were comparable to CCL21 stimulated M13HS-8 hybrid cells (Figure 2D). The finding that blockage of PI3K/AKT signaling resulted in a much higher inhibition of M13HS-8 cell migration than blockage of MAPK signaling might indicate that cell migration signals are predominantly processed via PI3K/AKT signaling in this hybrid cell line.

## Discussion

In the present study we investigated the migratory activity of M13HS-2 and M13HS-8 hybrid cell lines in response to the chemokine CCL21. Our data clearly show that both hybrid cell lines responded to CCL21 stimulation with an increased migratory activity indicating that fusion of CCL21 non-responding breast epithelial cells exhibiting stem cell characteristics and breast cancer cells can give rise to CCL21 responding hybrid cell lines.

The CCL21/CCR7 axis has been associated with lymph node metastasis of several cancers, including breast (for review see: [34]). Data of Cabioglu and colleagues provided evidence that CCR7 together with CXCR4 are valid biomarkers predicting axillary lymph node metastasis in T1 breast cancer [47]. More recent data indicated that in addition to CXCR4 and CCR7 and its ligands also EGFR was significantly higher expressed in breast tumor cells with lymph node metastasis, which was correlated to shorter survival period of afflicted patients [35]. The correlation between CCR7 expression and lymph node metastasis of tumor cells was further approved in animal studies. PyVmT mammary tumor cells expressing CCR7 preferentially colonized the lymph nodes, whereas CCR7 negative PyVmT breast cancer cells metastasized to lung tissue [48]. Likewise, stable expression of CCR7 in B16 melanoma cells resulted in a markedly increased lymph node metastasis as compared to empty vector transfected B16 melanoma cells [49]. The correlation between CCR7 expression and lymph node metastasis was further verified by administration of neutralizing anti-CCL21 antibodies that markedly blocked lymph node metastasis of CCR7 expressing B16 melanoma cells [49]. Data of Wiley and colleagues further indicate that CCL21, and not CCL19, preferentially mediates lymph node metastasis of cancer cells. This might be attributed to findings showing that CCL19 is predominantly expressed by lymphatic endothelial cells, whereas CCL21 is constitutively expressed on specialized high endothelial venules (HEVs) of lymph nodes, Peyer's patches, thymus, spleen and mucosal tissue [25], [26]. Moreover, several lines of evidence suggested that CCL19 and CCL21 have different properties on CCR7 activation and receptor internalization [28], [50]–[52]. In fact, CCL19, but not CCL21, induced a rapid time- and concentration dependent CCR7 internalization and desensitization, thereby decreasing the ability of CCR7 expressing cells to respond to a second stimulus [28], [50]. Dendritic cells migrated more efficiently towards CCL21 gradients albeit cells displayed similar sensitivities to both chemokines suggesting how CCL19 and CCL21 may signal differently to fine-tune dendritic cell homing and positioning within the lymphatic system [51]. Studies of dendritic cell homing into lymph node in CCL19 and CCL21 deficient mice revealed that a complete deficiency of CCL19 and CCL21, but not CCL19 alone, was found to be associated with abnormal frequencies and localization of dendritic cells in naïve lymph nodes [53]. Likewise, CCL19 was not required for dendritic cell migration from the skin, full dendritic cell maturation and efficient T lymphocyte priming suggesting that CCL21 is the critical CCR7 ligand regulating dendritic cell homeostasis and function *in vivo* [53], which may also belong to cancer metastasis.

The biological phenomenon of cell fusion has been linked to cancer progression. A plethora of data demonstrated that hybrid cells could exhibit tumor promoting properties such as an increased tumorigenic and metastatic behavior, an increased proliferation rate as well as an increased drug resistance [3]–[19].

However, cell fusion is associated with an increased level of aneuploidy and genetic instability (such as loss of chromosomes, translocation, deletions, amplification) in hybrid cells and each of these processes runs in a different manner in each evolving hybrid cell clone resulting in a unique hybrid cell phenotype [54]. Thus, cell fusion resembles Darwinian evolution and only the fittest hybrid cells will survive. Elderly cell culture experiments demonstrated that the survival rate of hybrid cells was solely around 1% [55], [56], which is in agreement with recent data of Wang and colleagues that estimated a cell fusion frequency of about 1.4% [22]. Moreover, Wang et al. were able to study the fate of single hybrid cells for several weeks [22]. Interestingly, hybrid cells firstly remain in a quiescent state for 4 weeks with no signs of cell division and about half of the hybrids perished within 6 weeks, possibly due to so-called mitotic catastrophe [22], [57]. In less than 5% of the remaining hybrids, cell division became conspicuous at 8 weeks, whereby most daughter cells showed mutually divergent morphologies and died [22].

Considering that the mean survival rate of hybrid cells is about 1 to 1.5% it can be concluded that a tumor of 1 cm^3^, approximately consisting of 1×10^9^ cells, should harbor about 1×10^5^ to 1.5×10^5^ proliferating hybrid cells. At a first glance, this cell number seems to be rather low. However, hybrid cells are proliferating, and even may possess an increased proliferation rate than normal tumor cells [6], thus the number of hybrid cells exhibiting novel properties is increasing permanently. Moreover, it should be emphasized that the fusion between tumor cells and other cells is not a one-time, but rather a permanent process. Tumor tissue resembles chronically inflamed tissue [58], [59] and (chronic) inflammation together with proliferatory conditions are two strong mediators of positively triggering cell fusion [60]. Thus it can be assumed that the frequency of cell fusion events should be increased in cancerous tissues, which should also have an impact on the number of surviving hybrid cells exhibiting novel properties.

The finding that M13HS hybrid cell lines possess a different phenotype and properties than their parental derivatives is in view with the hypothesis that cell fusion plays a crucial role in cancer progression [3], [6], [10], [13]. Goldenberg and colleagues reported about fusion events between hamster somatic cells and hamster tumor cells that occurred *in vivo* and which gave rise to stable hybrid cells that were uniformly lethal for its hamster host [7], [20]. Hybrid cells derived from fusion events between weakly malignant Cloudman S91 melanoma cells and murine mouse macrophages carried both mouse and human chromosomes and expressed human genes [17], [61]. Moreover, these hybrid cells also showed an increased motility *in vitro*, an enhanced metastatic potential *in vivo*, and also tended to be super melanotic [17], [61], [62]. Andersen and co-workers demonstrated that multiple myeloma patient-derived osteoclasts contained normal macrophage nuclei as well as multiple myeloma nuclei harboring chromosomal translocation suggesting that these cellular entities have originated by cell fusion [63]. The nuclei of myeloma B cell clone origin appeared fully integrated amongst the other nuclei and were transcriptionally active [63]. Because of that the authors concluded that fusion of osteoclasts and multiple myeloma cells leads to a reprogramming and/or transformation of these hybrid cells, thereby giving rise to highly efficient bone resorbing hybrid cells, which might be a possible explanation for the unrecognized mechanism of bone destruction in multiple myeloma patients [63]. By surgically joining a GFP mouse to an APC^Min/+^:ROSA26 mouse (a so-called parabiosis model), GFP and β-galactosidase double positive cells were identified in the transformed intestinal tissue of the APC^Min/+^:ROSA26 mouse indicating that cell fusion has occurred [14]. Isolation of these hybrid cells and subsequent transcriptome analysis revealed identity characteristics of both parental derivatives, but also showed a unique subset of transcriptomes including genes known to be modulated in metastasis [14] further suggesting that fusion between tumor cells and macrophages might be a unifying explanation for metastasis [13]. In addition to macrophages stroma cells and stem (-like) cells have also been identified as fusion partners for tumor cells. Data of Jacobsen et al. revealed that breast cancer epithelial cells can fuse with mouse stroma cells, thereby giving rise to highly malignant hybrid cells possessing an increased tumor initiation capacity [21]. Interestingly, hybrid cells do not only harbor both murine and human chromosome, but in a subset of hybrid cells (about 8%) also mouse/human chromosomal translocations were detected [21] that further contribute to the heterogenic character of hybrid cells. Recently, the spontaneous fusion of cancer-stromal cells was postulated as a possible mechanism of prostate cancer androgen-independent progression [22]. Cultivation of androgen-dependent LNCaP prostate cancer cells with cancer-associated prostate myofibroblast cells resulted in frequent fusion events and the origin of hybrid cells exhibiting genomic alterations concomitant with an androgen-independent phenotype [22]. Our own studies provided evidence that tumor cells could also fuse with stem cells or stem-like cells, thereby giving rise to hybrid cell lines exhibiting novel properties, like an altered migratory activity and/or an enhanced drug resistance [3]–[5], [23], [64]. M13MDA435 hybrid cell lines, which derived from spontaneous fusion events between M13SV1-EGFP-Neo epithelial cells exhibiting stem cell characteristics and the EGF non-responsive MDA-MB-435-Hyg breast cancer cells, responded to EGF stimulation with an increased migratory activity [5], [23]. Hybrid cell lines derived form spontaneous fusion events between murine BMDCs and 67NR-Hyg mouse mammary carcinoma cells possessed a markedly increased resistance towards doxorubicin, paclitaxel, etoposide and 17-DAMG as compared to their chemotherapeutic drug sensitive parental derivatives [64].

Both hybrid cells responded to the chemokine CCL21 with an increased migratory activity, which was different to the parental derivatives albeit they are positive for CCR7 and CCR7 signaling. Compared to HS578T-Hyg breast cancer cells and M13HS hybrids M13SV1-EGFP-neo breast epithelial cells exhibiting stem cell characteristics expressed markedly lower CCR7 levels, which might be an explanation for previous flow cytometry data showing virtually no CCR7 expression on M13SV1-EGFP-Neo cells [5]. Thus, surface expression of CCR7 on M13SV1-EGFP-Neo might be too low to be detected by conventional flow cytometry. Likewise, chemokine receptors are often stored in intracellular vesicles [65] and thus remain invisible if solely chemokine receptors located in the plasma membrane are stained. Nonetheless, stimulation of M13SV1-EGFP-Neo cells with CCL21 resulted in activation of PI3K/AKT and MAPK signaling further indicating functional CCR7 expression.

It is well recognized that chemokines can also bind to so-called atypical chemokine receptors (ACRs), which have also been termed decoy receptors or silent receptors due to their capability to support internalization and degradation of chemokines [66]. Likewise, most ACRs fail to activate signal transduction directing chemotaxis [66]; a matter that also applies to the CCL21 specific ACR CCRL1 (also named CCX-CKR) [67]. In fact, CCRL1 (CCX-CKR) did not couple to signaling pathways present in HEL293 cells used by most other chemokine receptors, such as G_αi_-containing heterotrimeric G-protein complexes [67]. Moreover, as reviewed by Comerford and colleagues CCX-CKR internalization depends on caveolins, which is different to the clathrin-coated pits and β-arrestin dependent internalization of conventional G-protein coupled receptors [68]. Thus, we conclude that the CCL21 observed effects in the investigated cell lines and hybrid cells lines truly depended on the interaction of CCL21 with its receptor CCR7.

The reason why both hybrid cell lines responded to CCL21 stimulation with an increased migratory activity, but not the parental cells albeit they displayed a functional CCR7 signaling as indicated by enhanced pMAPK^p42/44^ levels, remains ambiguous. Western Blot studies, calcium measurements and cell migration studies revealed a differential PLC-β/γ, PI3K/AKT and MAPK^p42/44^ signaling upon CCL21 stimulation among parental cell lines and hybrid cell lines. However, the CCL21 induced migration of both hybrid cell lines can not be simply attributed to the activation of a certain signal transduction pathway. For instance, both hybrid cell lines displayed a CCL21 mediated calcium influx, but inhibition of PLC-β/γ activity by U73122 only impaired the migration of M13HS-2 hybrid cells, whereas the migration of M13HS-8 hybrid cells remained unchanged. Likewise, both the spontaneous and CCL21 induced migration of M13HS-8 hybrid cells was effectively blocked by the PI3K inhibitor Ly294002, whereas in M13HS-2 cells only the CCL21 induced migration depended on PI3K/AKT signaling. The differential susceptibility of M13HS-2 and M13HS-8 hybrid cell lines towards Ly294003 inhibition might be explained to the higher basal pAKT levels in M13HS-8 hybrid cells, possibly attributed to autocrine and paracrine loops or a to differential regulation of AKT dephosphorylation. By contrast, M13SV1-EGFP-Neo breast epithelial cells exhibiting stem cell properties does not exhibit elevated basal pAKT levels even though the cells spontaneous migratory activity was significantly blocked by Ly294002.

Likewise, CCL21 mediated MAPK^p42/44^ phosphorylation was markedly enhanced in M13SV1-EGFP-Neo cells, M13HS-2 cells and M13HS-8 cells, but not in HS578T-Hyg cells. CCR7 dependent MAPK^p42/44^ activity is triggered via G protein-dependent and –independent mechanisms (for review see [27]). Thus, MAPK^p42/44^ activation in M13SV1-EGFP-Neo cells, M13HS-2 cells and M13HS-8 cells could be attributed to both G protein mechanisms. By contrast, in HS578T-Hyg breast cancer cells conceivably only one mechanism might be active. However, it can not be ruled out that lower MAPK^p42/44^ phosphorylation levels in HS578T-Hyg cells are additionally attributed to a differential kinetic of MAPK^p42/44^ dephosphorylation.

In addition to the differential activation and kinetics of CCR7 signal transduction cascades upon CCL21 stimulation Western Blot studies further suggest a differential activation and kinetics of signal transduction crosstalks in both hybrid cell lines and parental cell lines. Inhibition of CCR7 induced PI3K/AKT signaling and MAPK signaling with Ly294002 (PI3K inhibitor) and PD98059 (MEK inhibitor) demonstrated that Ly294002 also impaired the CCL21 induced MAPK^p42/44^ phosphorylation in HS578T-Hyg breast cancer cells, M13SV1-EGFP-Neo breast epithelial cells exhibiting stem cell properties and M13HS-2 hybrid cells. Several studies indicated a putative role of PI3K in MAPK^p42/44^ activation. For instance, COS cells activation by both endogenous ERK2 and RAS by low, but not high, EGF concentrations was suppressed by PI3K inhibitors [69]. Likewise, in T-cells and B-cells, respectively, MAPK^p42/44^ activation by TCR signaling and BCR signaling, respectively, is dependent on PI3K [70], [71]. Moreover, Lopez-Ilasaca et al. reported about a linkage of G protein-coupled receptors to the MAPK signaling pathway through class IB PI3Kγ and phosphotyrosine kinase (PTK), SHC, GRB2, SOS, RAS and RAF signaling [31]. Because Ly294002 effectively blocked MAPK phosphorylation in HS578T-Hyg breast cancer cells, M13SV1-EGFP-Neo breast epithelial cells exhibiting stem cell properties and M13HS-2 hybrid cells we assume that MAPK^p42/44^ activation in these cells also depends on PI3K. By contrast, in the M13HS-8 hybrid cell line Ly294002 did not impair MAPK^p42/44^ phosphorylation suggesting that in these cells PI3K signaling is not involved in MAPK^p42/44^ activation.

Western Blot analysis further revealed that PD98059 likely blocked AKT phosphorylation in M13SV1-EGFP-Neo cells and M13HS-8 hybrid cells suggesting that MEK/MAPK signaling modulates AKT phosphorylation. However, at present we do not have a suitable explanation for this finding. A crosstalk between MEK1 activity and PI3K/AKT signaling has recently been reported [72]. However, MEK1 was identified as an essential regulator of PTEN, through which it controls phosphatidylinositol-3-phosphate (PIP3) accumulation and AKT signaling [72]. MEK1 is necessary for PTEN membrane recruitment, thereby leading to PIP3 turnover concomitant with PI3K inhibition and decreased pAKT levels [72]. By contrast, blockage of MEK1 activity reduces PTEN membrane recruitment, increases PIP3 accumulation concomitant with AKT activation [72]. Thus, if such a crosstalk would be active in the investigated cells elevated pAKT levels should have been observed in PD98059 treated cells, which, however, was not the case. Nonetheless, decreased pAKT levels in PD98059 and PD98059 plus CCL21 stimulated cells were reproducible indicating a real effect. Thus, the mechanism how PD98059 impairs AKT phosphorylation remains to be elucidated.

The finding that M13HS hybrid cell lines differ in the kinetics of CCR7 signal transduction cascades and crosstalks is in view with data of Ozel et al. [23]. In this study, M13MDA435 hybrid cell lines were analyzed exhibiting a differential RAF-AKT crosstalk [23]. As a consequence, M13MDA435-1 and -3 hybrid cell lines showed a diametrically opposed effect to the PI3K inhibitor Ly294002: while the migration of M13MDA435-3 hybrid was markedly impaired, treatment of M13MDA435-1 hybrid cells with Ly294002 yielded in a significantly increased locomotory activity [23]. These data show that the kinetics of signal transduction cascades as well as crosstalks could vary among hybrid cell clones, which is in accordance to previous studies [3], [5], [17], [23] and which depict the random nature of cell fusion and subsequent origin of hybrid cells.

The differential phenotype of the M13HS hybrid cell lines among themselves as well as in comparison to their parental derivatives is most likely attributed to the fact that they originated from different parental cell types exhibiting a differential gene expression profile and epigenetic background. Lu and Kang demonstrated that hybrid cells, derived from highly specific MDA-MB-231 lung and bone metastatic variants, co-expressed lung and bone metastasis signature genes and were highly metastatic to both lung and bone suggesting a phenotypic overlap [73]. However, the authors argued that this phenotypic overlap was most likely attributed to the co-existence of regulatory epigenetic mechanisms from both fusion partners, thereby giving rise to hybrid cells expressing a dual set of organ-specific metastasis genes [73]. Miller and colleagues demonstrated that parental tumor cells, which were isolated from the same primary tumor can give rise to hybrid cells not only exhibiting a phenotypic overlap of parental properties, but do also possess novel characteristics [74]. In this work, highly tumorigenic and methotrexate resistance 168FAR tumor cells were fused with 44FTO cells that spontaneously metastasize and are 5-FU resistant [74]. Both parental cell lines were isolated from a spontaneously arising mammary tumor of a BALB/cFC3H mouse [74]. Hybrid clones showed a phenotypical overlap of parental properties, thus being tumorigenic, metastatogenic and resistant to both methotrexate and 5-FU [74]. However, hybrid cells also became resistant to melphalan, a compound to which both parental cell lines were sensitive for [74], indicating that cell fusion can give rise to hybrid cells exhibiting novel properties. In fact, in most studies reporting about tumor hybrid cells exhibiting novel properties tumor cells (were) fused with distinct cell types including macrophages [17], [61], [62], [75]–[77], stroma cells [21], [22], and stem (-like) cells [4], [5], [23], [64]. Due to the different phenotype of parental cells possessing a differential gene expression pattern, including normal mRNA and miRNA, as well as a differential epigenetic profile hybrid cells will originate each exhibiting a chaotic, but unique epigenetic/genetic reprogramming. Conjointly, transition from a heterokaryon status to a synkaryon status (fusion of single nuclei to one nucleus) is generally accompanied by chromosomal rearrangements and even losses that further drive the degree of aneuploidy and chromosomal instability in hybrid cells [10]. Moreover, data of Rappa and colleagues revealed that breast cancer/multipotent stromal cell hybrids underwent ploidy reduction and morphological reversal to breast carcinoma-like morphological characteristics, while maintaining a mixed breast cancer-mesenchymal expression profile [78]. Thus, ploidy reduction is another mechanism contributing to the origin of unique hybrid cells.

Cell fusion resembles Darwinian evolution and only the fittest hybrid cells/cell clones will survive. Due to the random nature of cell fusion, various hybrid cell clones will evolve. Even though they could respond similar to the same growth factor or chemokine, they could differ markedly in the overall intracellular processing of these extracellular signals. This knowledge has a great impact on the understanding of how to treat cancer, particularly if specific receptors, kinases, and signal transduction pathways have been chosen as targets.

In summary, our data provide evidence that cell fusion could give rise to hybrid cells exhibiting altered properties. Here the fusion of two CCL21 non-responding parental derivative gave rise to highly CCL21 sensitive hybrid cell lines. Because the CCL21/CCR7 axis has been associated with lymph node metastasis formation of breast cancer we conclude that cell fusion between breast cancer cells and breast epithelial cells exhibiting stem cell properties is a possible mechanism how metastatic cancer hybrid cells can originate.

## Supporting Information

Figure S1 **Relative AKT, pAKT, MAPK, pMAPK levels.** Relative intensities of native and phosphorylated proteins were determined in relation to the housekeeping gene elf4E, whereby untreated cells (con) served as a control and were set to 100%. **(A)** HS578T-Hyg breast cancer cells **(B)** M13SV1-EGFP-Neo breast epithelial cells exhibiting stem cell characteristics, **(C)** M13HS-2 hybrid cells, **(D)** M13HS-8 hybrid cells. Shown are the mean±SD of n = 3 independent experiments. Densitometric analysis of Western Blot data was performed by using the ImageJ software application. Statistical significance was calculated using Student *t*-Test, whereby p<0.05 was considered as significant. *  =  statistical significance in relation to control; †  =  statistical significance in relation to 5 min CCL21 stimulation.
(TIF)
