# Supplementary figures and images for: Fusion of CCL21 Non-Migratory Active Breast Epithelial and Breast Cancer Cells Give Rise to CCL21 Migratory Active Tumor Hybrid Cell Lines
Source: PLoS One. 2013 May 7;8(5):e63711. doi: 10.1371/journal.pone.0063711 (PMC3646822; doi:10.1371/journal.pone.0063711)

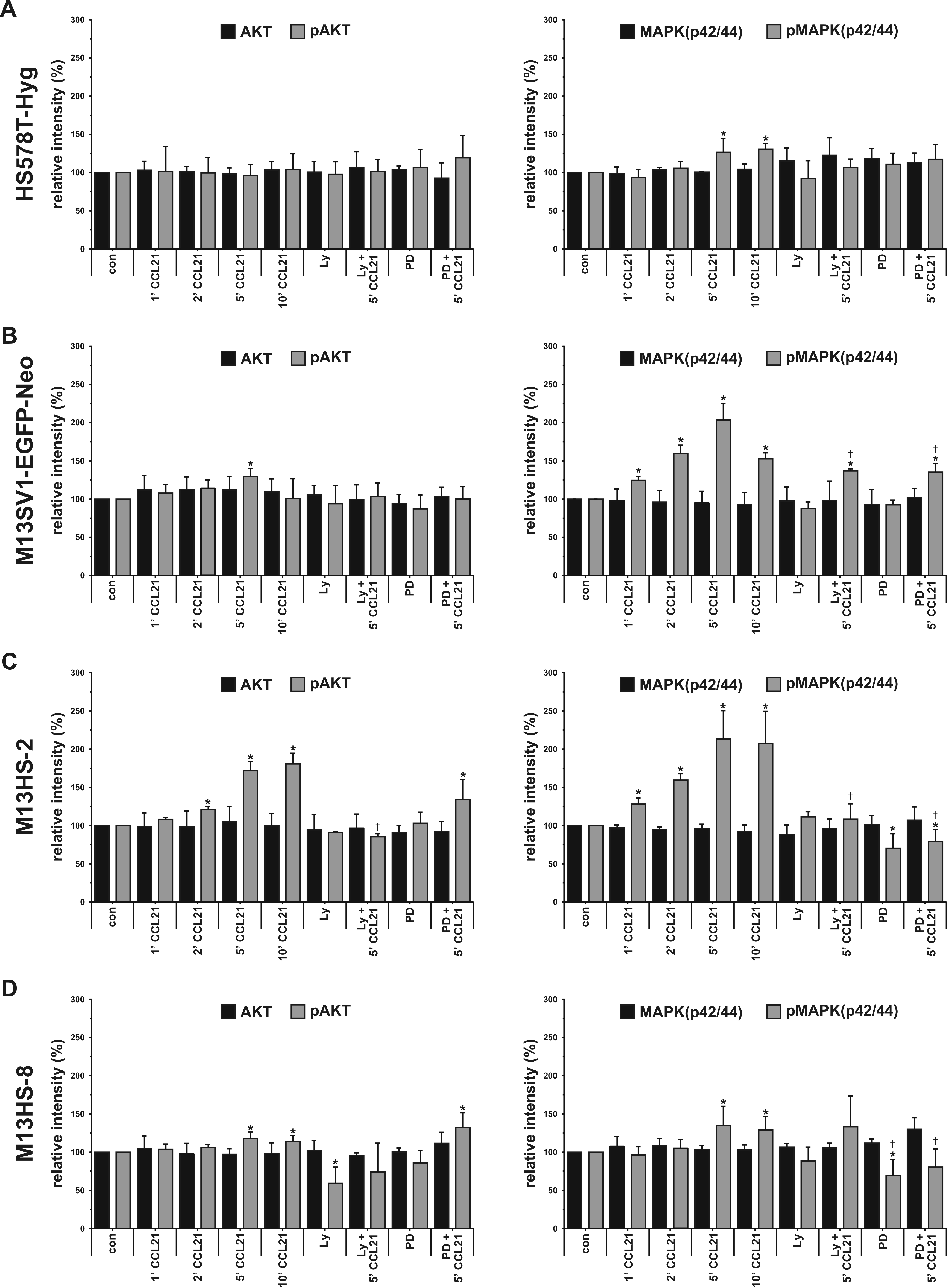

Supplement: Figure S1 — Relative AKT, pAKT, MAPK, pMAPK levels. Relative intensities of native and phosphorylated proteins were determined in relation to the housekeeping gene elf4E, whereby untreated cells (con) served as a control and were set to 100%. (A) HS578T-Hyg breast cancer cells (B) M13SV1-EGFP-Neo breast epithelial cells exhibiting stem cell characteristics, (C) M13HS-2 hybrid cells, (D) M13HS-8 hybrid cells. Shown are the mean±SD of n = 3 independent experiments. Densitometric analysis of Western Blot data was performed by using the ImageJ software application. Statistical significance was calculated using Student t-Test, whereby p<0.05 was considered as significant. * = statistical significance in relation to control; † = statistical significance in relation to 5 min CCL21 stimulation. (TIF) [file pone.0063711.s001.tif]
